# Sensitivity of marine fish thermal habitat models to fishery data sources

**DOI:** 10.1002/ece3.7817

**Published:** 2021-09-08

**Authors:** Laura Nazzaro, Emily Slesinger, Josh Kohut, Grace K. Saba, Vincent S. Saba

**Affiliations:** ^1^ Center for Ocean Observing Leadership Department of Marine and Coastal Sciences School of Environmental and Biological Sciences Rutgers, The State University of New Jersey New Brunswick NJ USA; ^2^ National Oceanic and Atmospheric Administration National Marine Fisheries Service Northeast Fisheries Science Center Geophysical Fluid Dynamics Laboratory Princeton University Princeton NJ USA

**Keywords:** black sea bass, generalized additive models, marine fisheries data sources, thermal habitat models, U.S. Northeast Shelf

## Abstract

Statistical models built using different data sources and methods can exhibit conflicting patterns. We used the northern stock of black sea bass (*Centropristis striata*) as a case study to assess the impacts of using different fisheries data sources and laboratory‐derived physiological metrics in the development of thermal habitat models for marine fishes. We constructed thermal habitat models using generalized additive models (GAMs) based on various fisheries datasets as input, including the NOAA Northeast Fisheries Science Center (NEFSC) bottom trawl surveys, various inshore fisheries‐independent trawl surveys (state waters), NEFSC fisheries‐dependent observer data, and laboratory‐based physiological metrics. We compared each model's GAM response curve and coupled them to historical ocean conditions in the U.S. Northeast Shelf using bias‐corrected ocean temperature output from a regional ocean model. Thermal habitat models based on shelf‐wide data (NEFSC fisheries‐dependent observer data and fisheries‐independent spring and fall surveys) explained the most variation in black sea bass presence/absence data at ~15% deviance explained. Models based on a narrower range of sampled thermal habitat from inshore survey data in the Northeast Area Monitoring and Assessment Program (NEAMAP) and the geographically isolated Long Island Sound data performed poorly. All models had similar lower thermal limits around 8.5℃, but thermal optima, when present, ranged from 16.7 to 24.8℃. The GAMs could reliably predict habitat from years excluded from model training, but due to strong seasonal temperature fluctuations in the region, could not be used to predict habitat in seasons excluded from training. We conclude that survey data source can greatly impact development and interpretation of thermal habitat models for marine fishes. We suggest that model development be based on data sources that sample the widest range of ocean temperature and physical habitat throughout multiple seasons when possible, and encourage thorough consideration of how data gaps may influence model uncertainty.

## Graphical abstract

This study assesses the sensitivity of generalized additive models, frequently used in habitat modeling, to various fisheries data sources used as input by using black sea bass (*Centropristis striata*) as a case study. Results show that similarities and differences between habitat models can inform uncertainty estimates and can have strong implications for coupled oceanographic habitat models often used to represent spatial habitat distributions for a wide variety of applications.

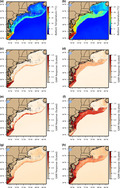

## 1 INTRODUCTION

Defining marine habitat indicators is challenging due to the complex interaction between marine species and environmental variability, but various methodologies such as process‐based laboratory experiments (Slesinger et al., 2019) and empirical field studies (Cullen & Guida, 2021; Kleisner et al., 2017; McHenry et al., 2019) have been developed to determine the relationships between marine ectotherms and the environment. Such relationships can be developed because metabolic processes in ectotherms are tightly coupled to environmental temperatures (Clarke & Johnston, 1999; Verberk et al., 2016) and they are more vulnerable to warming temperatures than their terrestrial counterparts (Pinsky et al., 2019). Understanding how living marine resources respond to changes in environmental conditions is useful for management and policy decisions in a rapidly changing climate, as well as anticipating potential species shifts and introductions/evacuations in a local ecosystem. A common response to ocean warming includes rapid distribution shifts, usually poleward, for many marine species (Kleisner et al., 2016; Nye et al., 2009), which can lead to management conflicts and changes in species compositions in newly occupied regions (Pinsky et al., 2018). While laboratory and empirical studies have proven to be useful in elucidating these range shifts, they are also subject to many limitations. Laboratory studies are conducted in controlled environments to promote replication under optimal conditions whereby the fish are fed ad libitum, predators are absent, and interactive effects from other stressors are removed, sometimes resulting in skewed thermal optima (Jutfelt et al., 2018; Slesinger et al., 2019). Environmental response models for a single species developed in empirical field studies, on the other hand, can look quite different from one another depending on the specific type of model and training datasets used, leaving many models vulnerable to misinterpretation or bias (Bahn & McGill, 2013). These methodological issues are concerning because one of the overarching goals in habitat model development is to provide a tool to predict future distributions under various climate change scenarios. While they are incredibly useful and effective tools, the limitations are often glossed over rather than used to inform key areas of uncertainty.

The Mid‐Atlantic Bight (MAB), located within the U.S. Northeast Shelf, provides a natural laboratory to investigate the impacts of fishery data source on thermal habitat models because this region has exceptionally high seasonal temperature variability and substantial long‐term ocean warming (Chen et al., 2020), and offers long‐term datasets of fishery‐independent and dependent data to build thermal habitat models for many marine taxa. The MAB spans from Cape Hatteras, North Carolina to Cape Cod, Massachusetts, with a broad shelf that transitions to a steep slope ~150 km from shore. Ectotherms that inhabit the MAB have distributions and life histories coupled to ocean temperature and thus many species undergo seasonal migrations in which they move between nearshore habitat during the summer, often for spawning, and southern or warmer offshore shelf‐slope habitats during cooler months of the year (e.g., butterfish; Cross et al., 1999; black sea bass: Drohan et al., 2007; summer flounder: Packer et al., 1999; bluefish: Shepherd & Packer, 2006; scup: Steimle et al., 1999). On longer timescales, several marine species have already shifted their distribution poleward and/or into deeper waters (Kleisner et al., 2016; Nye et al., 2009) and are projected to continue to shift under continued ocean warming (Kleisner et al., 2017; McHenry et al., 2019; Morley et al., 2018). If we hope to understand short‐ and long‐term species distribution shifts in such a highly dynamic region, it is important to accurately interpret the relationships between those species distributions and the surrounding environment.

Within the MAB, black sea bass (*Centropristis striata*) are an ideal study species to test the effects of varying data sources on model shape used for the development of thermal habitat models due to the abundance of existing data and research on black sea bass. Black sea bass are an abundant demersal finfish often found near rock and artificial reefs (Drohan et al., 2007) that support lucrative recreational and commercial fisheries in the MAB (NEFSC, 2017). The U.S. Northern stock of black sea bass occupies waters north of Cape Hatteras, NC (Roy et al., 2012), inhabiting shallow inshore water during the summer and migrating offshore during the late fall to overwinter at the shelf‐slope front (Moser & Shepherd, 2008). Like many species, black sea bass have shifted their distribution northward along the U.S. northeast coast as ocean temperature has warmed over the last few decades (Bell et al., 2015; Kleisner et al., 2016). Multiple black sea bass thermal habitat models have been developed using fisheries data paired with in situ and modeled environmental parameters. For example, Kleisner et al. (2017) and McHenry et al. (2019) each developed GAMs by associating presence‐absence data with several environmental covariates. While similar, the two models used different data sources as input to their respective models, resulting in some key differences in the shape of the response curves of the GAMs. Differences in model output describing the relationships of bottom temperature to black sea bass habitat can have significant implications for both our current interpretation of temperature as it applies to fish distribution (e.g., optimal temperatures) and for predicting future thermal habitat under varying climate scenarios. While differences between various models are not inherently a problem, examining the reasons behind those differences can improve our understanding of the models themselves and can include where and why there is more (or less) uncertainty in each model, and what types of applications different models may be most appropriately suited to rather than opting for a “one size fits all” approach. Altogether, this analysis could provide a framework for using these models to predict thermal habitat.

Here we show that thermal response curves developed using GAMs vary by model type and data source. Specifically, we assessed field‐based measurements from fishery‐independent and fishery‐dependent data sources as they relate to measured and modeled bottom temperature, and in situ laboratory‐derived thermal optima from physiological studies (Slesinger et al., 2019). We limited our models to habitat based on bottom temperature because it allowed us to directly compare empirical models derived from survey data to recent process‐based laboratory data that only measured the effects of temperature, and because bottom temperature has a well‐established physiological relationship with fitness for many demersal species, including black sea bass (Bicego et al., 2007; Clarke & Johnston, 1999). As a standard ocean variable resolved in many global and regional hydrodynamic hindcasts, nowcasts, and long‐term climate projections, habitat models based solely on temperature can be coupled to a wide variety of hydrodynamic simulations. Our multi‐model analysis provides new information on uncertainty, interpretation, and application of habitat models that is relevant to many marine species.

## 2 METHODS

Following Kleisner et al. (2017) and McHenry et al. (2019), we associated black sea bass presence‐absence data with environmental covariates, which we limited to bottom temperature in order to better compare models developed from different training data sources. We used the “mgcv” package in R version 3.6.1 (Wood, 2017) to develop presence–absence GAMs with a binomial distribution from five fisheries data sources paired with both measured and modeled bottom temperatures in order to determine whether GAM models developed using modeled temperatures were comparably effective to those developed with measured temperatures. The shape and performance of the curves were compared to each other and to the experimental physiological response curve from Slesinger et al. (2019) to assess compatibility between model results obtained via statistical models versus in situ laboratory data.

### 2.1 Fisheries data sources

We used data from four fisheries‐independent bottom trawl sources (Sections 2.1.1–2.1.4): a shelf‐wide federal bottom trawl survey, a shallow inshore federal bottom trawl survey spanning the entire MAB coast, and inshore state government bottom trawl surveys by New Jersey and Connecticut. All fisheries‐independent surveys followed a stratified random sampling design. Fisheries‐dependent observer data from a regional observer program was used as well (Section 2.1.5). Where available, data from 1985 through 2015 were used for this analysis (Table 1). We started with 1985 because it was the earliest year included in the decadal climatologies used for bias‐correcting that had full overlap with the hydrodynamic model used, and all surveys except one have data going back to 1989 or earlier, and thus, long‐term temporal coverage was similar. Data past 2015 are excluded because it is the final year covered by the hydrodynamic model. All survey methods only included juvenile and adult fish.

**TABLE 1:** Summary information of sampling by fisheries data sources used in this analysis

Survey	Years	Months sampled	Latitudinal range (°*N*)	Depth range (m)	Environmental data sampled	*N* (% samples w/black sea bass)
NEFSC	1985–2015	2–6,9–12	33.5–44.75	10–350	depth, surface and bottom temperature, surface and bottom salinity	20,690 (10.4%)
NEAMAP	2007–2015	4–6,9–11	35.1–41.5	5–60	depth, bottom salinity, bottom temperature, dissolved oxygen concentration, and saturation	2,570 (33.5%)
NJDEP	1988–2015	1,4,6,8,10 (other months included in early data)	38.5–40.5	5–40	depth, surface and bottom temperature, surface and bottom salinity, surface, and bottom dissolved oxygen	5,105 (39.3%)
CTDEEP	1985–2015	5–7,9–11	40.8–41.4	5–60	depth, surface and bottom temperature, surface, and bottom salinity	5,850 (17.9%)
Observer	1989–2015	1–12	35–44.5	10–250	*n*/a	190,303 (9.8%)

#### 2.1.1 National Marine Fisheries Service Northeast Science Center (NMFS NEFSC) bottom trawl survey

The NEFSC has run annual spring and fall bottom trawl surveys along the shelf from Cape Hatteras, NC to the Canadian Scotian Shelf since 1968. Most of the spring survey trawls occurred from March through mid‐May, and most fall survey trawls from September through mid‐November. Nets were towed throughout the 24‐hr day over the bottom at about 3.5 knots for 20–30 min. In 2009, coinciding with a transition in research vessels, the minimum survey depth was increased from 10 m to 20 m. Most spring samples were observed between 5 and 15℃ with salinity between 32 and 36 PSU; fall samples had a similar salinity range but a wider temperature range between ~5 and 25℃. More details on the survey design can be found in Azarovitz (1981). The gear on the research vessel being used since 2009 has higher catchability compared to the gear used before 2009, particularly for smaller fish (Miller et al., 2010), but for this analysis we consider that difference to be minimal compared to the differences in survey design and timing, particularly for presence–absence models as used in this study.

#### 2.1.2 Northeast Area Monitoring and Assessment Program (NEAMAP) bottom trawl survey

NEAMAP has conducted a shallow‐water bottom trawl survey spanning from Cape Hatteras, NC to Cape Cod, MA during the spring and fall since 2008 (Bonzek et al., 2008). Nets were trawled along the bottom during daylight at about 3.1 knots for 20 min in water up to approximately 20 m deep or 40 m in the sounds. The majority of spring survey samples were collected between mid‐April and mid‐June, and the majority of fall survey samples were collected from mid‐September through mid‐November. Both seasons sampled a narrow band of temperature, ~5–10℃ in spring and 15–20℃ in fall, across a comparatively wider salinity band, approximately 28–34 PSU.

#### 2.1.3 New Jersey Department of Environmental Protection (NJDEP) bottom trawl survey

The NJDEP Department of Fish and Wildlife has been conducting a week‐long ocean stock assessment bottom trawl survey five times a year from Sandy Hook, NJ to Cape Henlopen, DE since 1988 (Celestino et al., 2014). Year‐round, this survey sampled a narrow salinity band around 31–33 PSU across a very wide range of temperatures from 2 to 25℃.

#### 2.1.4 Connecticut Department of Energy and Environmental Protection (CTDEEP) Long Island Sound bottom trawl survey

The CTDEEP has been conducting bottom trawl surveys within the Long Island Sound three times each spring and twice each fall since 1984 (Gottschall & Pacileo, 2018). Sampling covered a wide range of temperatures from ~3–23℃ in water between 26 and 30 PSU.

#### 2.1.5 Northeast Fisheries Observer Program (NEFOP)

NEFOP is the regional component of the national observer program, managed by the NMFS NEFSC Fisheries Sampling Branch, that uses trained federal observers to collect catch data during commercial fishing trips (NMFS, 2019). We obtained data for the MAB shelf and Gulf of Maine from 1989 through 2015. While several gear types were included in the dataset, we limited our analysis to four types of bottom otter trawls (fish, scallop, twin, and Ruhle) to provide comparable data to the federal and state trawl surveys. Even after eliminating several gear types, this dataset offered an order of magnitude more samples than any of the fisheries‐independent surveys. The majority of the sampling was performed year‐round at the shelf break and canyons, as well as the edges of Georges Bank. As a fisheries‐dependent dataset, sampling locations were not randomly selected although black sea bass were not necessarily the target fishery for most samples.

### 2.2 Ocean bottom temperature sources

Fisheries data samples were paired with bottom temperature measured in situ with each trawl (where available) and bias‐corrected modeled bottom temperature (described below). Each of the four fisheries‐independent surveys collected oceanographic data for each survey sample by lowering a temperature probe to near‐bottom immediately before or after each tow was performed, so both in situ measured and bias‐corrected modeled bottom temperatures were available for trawls from these surveys. There were no in situ temperature data available from the commercial fishery observer program, and thus, presence–absence data were matched only to bias‐corrected modeled bottom.

We paired each tow from all surveys with bias‐corrected monthly averaged bottom temperatures from a Regional Ocean Modeling System (ROMS) non‐data‐assimilative hindcast simulation spanning 1981–2015. The simulation was an updated version of the one used in Kang and Curchitser (2015) with a newer Jerlov water type array, covering the entire Northwest Atlantic including the path of the Gulf Stream from the Gulf of Mexico to the northwest Atlantic including the Gulf of Saint Lawrence, with a horizontal resolution of 7 km and 40 terrain‐following vertical levels. Bottom bathymetry for the model was derived from the Shuttle Radar Topography Mission (SRTM) database (Farr et al., 2007), initial ocean boundary conditions from reanalysis data of Simple Ocean Data Assimilation (SODA 3.3.1) (Carton et al., 2018), and atmospheric forcing from the Drakkar forcing set (DFS 5.2) (Dussin et al., 2016). There was a persistent bottom temperature bias of around 2℃ across the shelf (Figure S1), which we corrected using a regional climatology.

The NOAA Northwest Atlantic climatology (Seidov et al., 2016) at a monthly temporal resolution and 0.1° spatial resolution was used to bias‐correct ROMS bottom temperatures. Several decadal climatologies, calculated using all data that was available in the World Ocean Database in 2013, are available, including three that overlapped with our time period: 1985–1994, 1995–2004, and 2005–2012. Climatology temperatures were provided at set depths of varying vertical resolution and we defined bottom temperature as the deepest point within the data. Vertical resolution of the climatology varied as a function of total water depth in the following ranges: 5 m resolution (0 m to 100 m depth), 25 m resolution (100 to 500 m), and 50 m resolution (>500 m). Thus, across the shelf, the matched bottom temperatures were within 5 m of true bottom. In the slope water, deeper than 100 m, bottom temperatures could have been up to 25 m shallower than actual bottom and averaged over a wider vertical swath, but deep water temperatures vary much less with small changes in depths than water temperatures closer to the surface. We averaged the monthly ROMS bottom temperatures over the corresponding decadal ranges, binned to the same grid as the climatology, and subtracted the climatology to determine a spatially varying bias to apply to each month for all years within the corresponding decade. The bias correction based on the 2005–2012 climatology was used for all years between 2005 and 2015. Individual trawls from all five surveys were paired with the nearest monthly averaged, bias‐corrected ROMS bottom temperature.

### 2.3 Model development and comparison

We used the generalized additive models (GAM) function from the R package mgcv 1.8–28 (Wood, 2017) to model presence–absence as a function of bottom temperature from the sources listed above. Separate GAMs, with thin plate regression spines and smoothing penalties modified to penalize null space, were created for each survey paired with in situ measured bottom temperature and another paired with bias‐corrected ROMS bottom temperature, except for observer data which had no measured bottom temperature available. GAM response was considered positive where the log‐odds prediction was greater than 0, and thermal optima, only defined for curves with a steady increase followed by a steady decrease, were considered to be the temperature at which the prediction reached its highest value.

For each GAM, we compared a traditional random Monte Carlo cross‐validation with 100 iterations of the model run using 75% of the data used to train the model and the remaining 25% withheld for testing. To account for some of the spatial and temporal autocorrelation and determine the reliability of the model when used to predict years that were not included in training, we compared grouped iterations for rolling consecutive 7‐year sets of trawls (approximately 25% of the data) withheld for testing while the remaining data were used for training. We used area under the receiver operating curve (AUC) as our cross‐validation metric to evaluate the predictive ability of the presence–absence model. AUC varies between 0 and 1, with better‐performing models scoring closer to 1, models with no predictive value scoring 0.5, and scores near 0 indicating models that predict the opposite of what is observed. We required all iterations of the cross‐validation to fall above an AUC of 0.6 in order to consider a model as having predictive value. To compare how well each model predicted data from other surveys compared to the training survey, we also compared AUC for each set of survey data based on each well‐described survey GAM.

In laboratory experiments conducted on black sea bass, Slesinger et al. (2019) measured standard and maximum metabolic rate to derive aerobic scope at a range of temperatures (12–30℃). The laboratory‐derived model from Slesinger et al. (2019) used aerobic scope as a dependent variable using a 3rd degree polynomial fit. In order to provide a laboratory‐derived model comparable to the presence–absence GAMs from the fishery‐independent and dependent data sources, we developed a laboratory data‐based Poisson family GAM for aerobic scope as a function of temperature, with a smoothing factor of 3.

AUC is an effective, quantitative metric that can be used to directly compare presence–absence species models. However, because laboratory data modeled aerobic scope and thus were not directly comparable to the GAMs predicting presence–absence the same metric could not be used to evaluate the performance of the laboratory model as it compared to the others. In order to do so, we performed additional cross‐validations for the laboratory dataset and the NEFSC, NJDEP, and observer survey datasets similar to the method in NEFSC (2014), but adapted for use with presence–absence data: For 100 iterations in a random Monte‐Carlo, the GAM based on the training dataset was used to predict the response of the testing dataset, which we then normalized to the maximum predicted response so all responses were scaled from zero to one. The survey iterations were randomized as described for the initial Monte‐Carlo described above, and the laboratory GAM iterations were randomized by selecting 9 samples of each temperature record (from originally 10–12 samples for each of five temperatures between 12 and 30℃) and then compared to testing data from the three surveys well‐described by temperature (NJDEP, NEFSC, and observer). Presence–absence survey GAMs were all asymptotic near zero for low temperatures, while aerobic scope was on a much larger scale and required scaling from 0 to 1 over the minimum to maximum range of response from 0 to 30℃ in order to be comparable. This scaled response value was calculated for each trawl in each of the 100 iterations and then converted to a habitat quality ranking between 1 (poorest quality) and 10 (highest quality) depending on this scaled response value (i.e., scaled GAM response between 0.0–0.1 was given a quality ranking of 1, scaled responses 0.1–0.2 a quality ranking of 2,… scaled response 0.9–1.0 a quality ranking of 10). For this version of the cross‐validation, the proportion of trawls that caught black sea bass in each of the ten categories was calculated and pattern of the proportion of trawls with black sea bass versus habitat quality ranking was used to assess model performance. Better model performance was indicated by increasing black sea bass catch with habitat quality ranking, regardless of the specific proportion of tows containing black sea bass.

## 3 RESULTS

### 3.1 Fisheries data coverage

**FIGURE 1:**
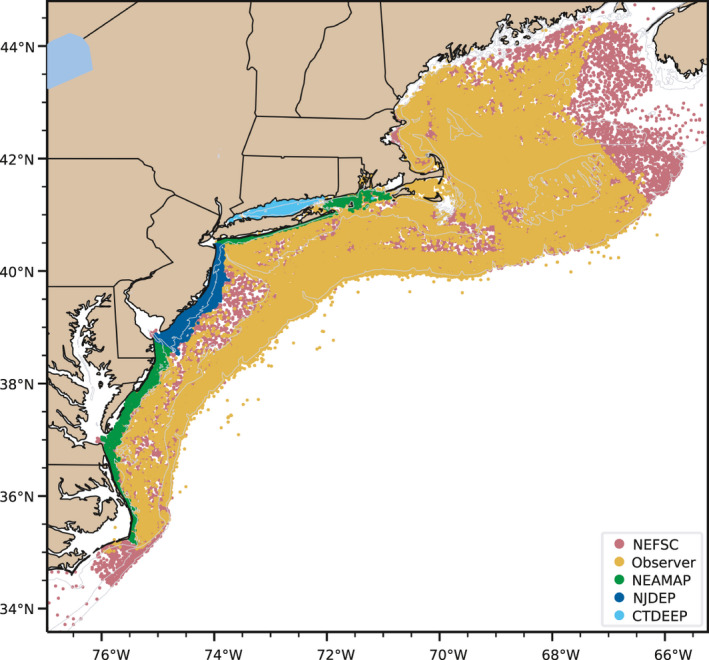
All sampling locations included in this study

Each of the data sources considered in this study provided thousands of samples within the MAB (Table 1, Figure 1), but had different temporal and spatial coverage. The NEFSC survey only sampled during the spring and fall seasons (Figure S2a,c), but covered a wide range of temperatures and a few distinct water masses across the entire shelf (Figure S2e‐g), and captured the seasonal black sea bass migration as a predominantly shallow‐water catch during the fall survey versus a largely shelf break catch during the spring survey (Figure S2b,d). The NEAMAP survey sampled a wide latitudinal range in shallow water during the same seasons but with slightly different timing and sampling pattern, following the seasonal transition from south to north from April into June, and from north to south from September into November (Figure S3a‐d) as opposed to the NEFSC survey which samples earlier in the spring (Figure S2a,c) and follows the same sampling pattern during each seasonal survey. Because of this, NEAMAP only sampled a relatively small range of temperatures, most of which lay inside an approximately 2℃ range for each season (Figure S3e‐g). The NJDEP survey covered a much smaller spatial area (Figure S4a), but had greater temporal coverage throughout the year. While it did not sample during every single month, the survey did sample during each season at least once, evenly covering the entire sampling area during each survey and including a wide range of temperatures throughout the year (Figure S4e‐g). The CTDEEP Long Island Sound survey covered a wide range of temperatures as well (Figure S4b‐d), but data from this survey were similarly limited in monthly temporal coverage and almost completely surrounded by land and therefore isolated from the rest of the shelf (Figure S4a). Of the five fisheries data sources, only the observer program provided consistent year‐round coverage. Sampling was concentrated in key fishing areas such as the canyons and the shelf break front (Figure S5a) but was relatively consistent throughout the year. Black sea bass caught in the surveys generally followed expected seasonal migration patterns, mainly inshore during the summer and transitioning into deeper water in the fall until the winter and spring when most of the catch was at the shelf break (Figure S5b).

### 3.2 Black sea bass GAM thermal response curves

**FIGURE 2:**
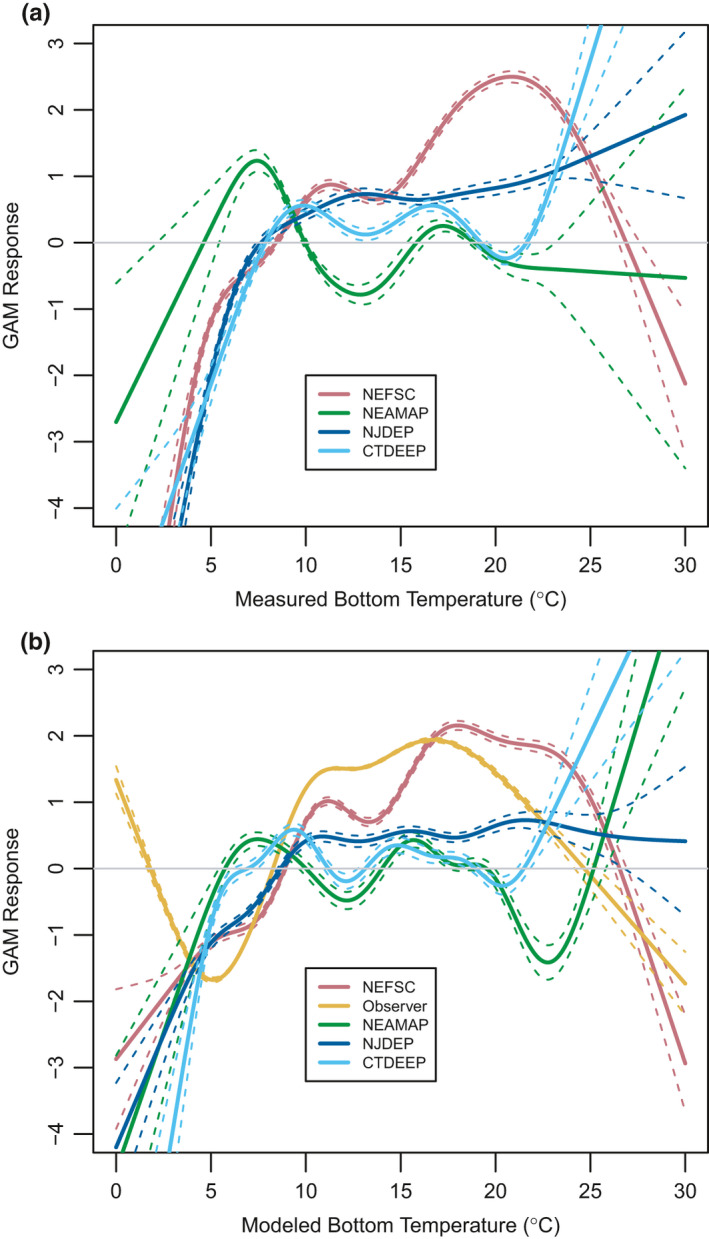
GAM log‐odds response ±1 *SE* for data from each survey as a response to in situ measured bottom temperature (a) and monthly averaged bias‐corrected ROMS bottom temperature (b)

Only GAMs based on NEFSC trawl survey and observer data exhibited a clear peak in thermal response, and both were equally well‐described with approximately 15% deviance explained. While the patterns varied slightly, they were generally the same with a positive response between approximately 8.5℃ and 25℃. The thermal optimum (*T*_opt_), the temperature at which the GAM peaked, was slightly warmer for the NEFSC data than it was for the observer data (18℃ vs. 16.7℃ ROMS bottom temperature). The NEFSC data resulted in a slightly bimodal GAM, with a weak and narrow spring peak in cool temperatures as well as a wider and stronger peak in warmer fall waters, as opposed to the smoother curve created by the year‐round observer data (Table 2, Figure 2). This is similar to the partial GAM including only bottom temperature from the McHenry et al. (2019) model that also used fisheries‐dependent observer data (Figure S6b).

**TABLE 2:** Performance of GAMs for each survey, and key descriptive characteristics for each curve including optimal temperature and range of temperatures exhibiting a positive response

Survey	Deviance explained	*T*_opt_	Positive response temperature range
NEFSC	16.5% (in situ) 15% (ROMS)	20.8℃ (in situ) 18.0℃ (ROMS)	8.5–26.8℃ (in situ) 8.9–26.5℃ (ROMS)
NEAMAP	4% (in situ) 2.6% (ROMS)	n/a	n/a
NJDEP	10.8% (in situ) 7% (ROMS)	n/a	7.7℃ and higher (in situ) 8.7℃ and higher (ROMS)
CTDEEP	4.5% (in situ) 2.2% (ROMS)	n/a	n/a
Observer	15.3% (ROMS)	16.7℃ (ROMS)	8.3–24.6℃ (ROMS)

The GAM response derived from NJDEP data increased toward higher temperatures before plateauing without reaching a natural peak or high‐temperature decrease. Despite the difference in shape, the lower thermal limit from this curve is remarkably similar to the lower limit in the NEFSC and observer data, at 7.7℃ for in situ bottom temperature and 8.7℃ for ROMS bottom temperature (Table 2). The GAM based on in situ temperature performed better than the GAM based on ROMS bottom temperature (10.8% deviance explained by in situ temperature versus 7% explained by ROMS temperature) (Table 2, Figure 2), but both explained less deviance when compared to the NEFSC data which had approximately 15% deviance explained by both temperature sources. The shape of the NJDEP curve was more similar to the partial GAM based on bottom temperature only from the Kleisner et al. (2017) model compared to the peaked curve seen in the NEFSC, observer (Figure 2), and McHenry et al. (2019) models (Figure S6a). The GAM based on laboratory data was somewhere between the two curves, with a gradual rise at low temperatures and minimal decrease at high temperatures. A peak in the laboratory‐based data existed but was not as strong as the other peaks and was a few degrees warmer than survey‐based GAMs at 24.8℃ (Figure S6c).

The remaining two fisheries data sources both performed poorly (<5% deviance explained) with erratic response curves (Table 2, Figure 2). For the NEAMAP‐based GAM, this was likely due to the narrow range of temperatures sampled (Figure S3), while for the CTDEEP‐based GAM it was more likely attributed to the isolated nature of Long Island Sound (Figure S4). Due to the poor performance of these models, they were excluded from the cross‐validation.

### 3.3 Cross‐validation

**FIGURE 3:**
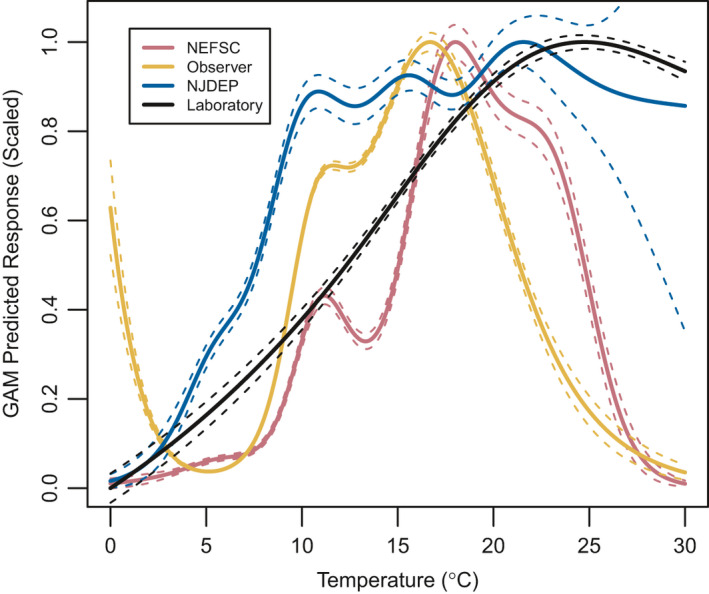
GAM response as a function of temperature, scaled from 0 to 1 based on all data for each dataset (±1 *SE*). These curves are used for the habitat quality ranking cross‐validation in Figure 6 and the habitat projections in Figure 7. NEFSC, observer, and NJDEP GAMs are based on ROMS bias‐corrected bottom temperature, while the laboratory GAM is based on measured temperature

Because the GAMs based on NEFSC and NJDEP survey data were similar when fitted to in situ bottom temperature and ROMS bottom temperature (Figure 2), and no in situ data were available for the observer dataset, cross‐validations were only performed using ROMS‐based models and temperatures. The scaled predictive response curves for each survey based on the full dataset that were used for the habitat quality ranking cross‐validation are shown in Figure 3.

**FIGURE 4:**
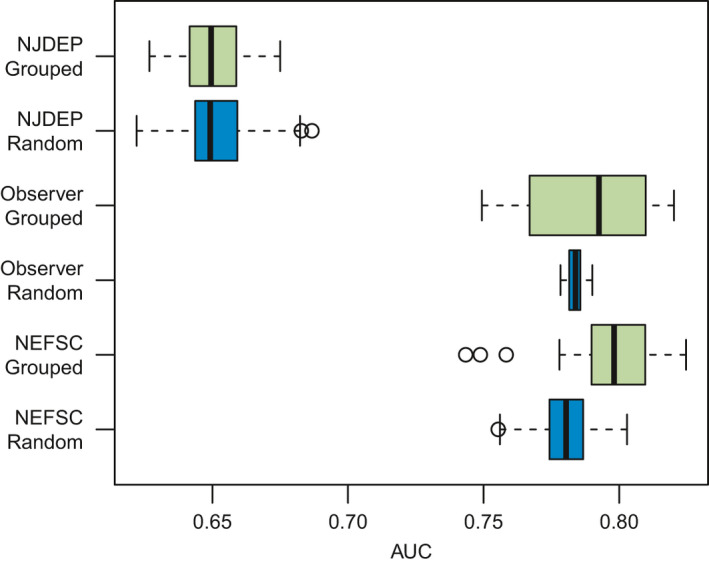
Boxplot of AUC cross‐validations metrics for the three well‐performing surveys (NJDEP, observer, and NEFSC). AUC values near 0.5 indicate models no better than random prediction, and closer to 1 indicating highest performance. Blue boxplots show the range of AUC values for the random cross‐validations, compared to the grouped cross‐validation with consecutive groups of years removed for testing shown in green

**FIGURE 5:**
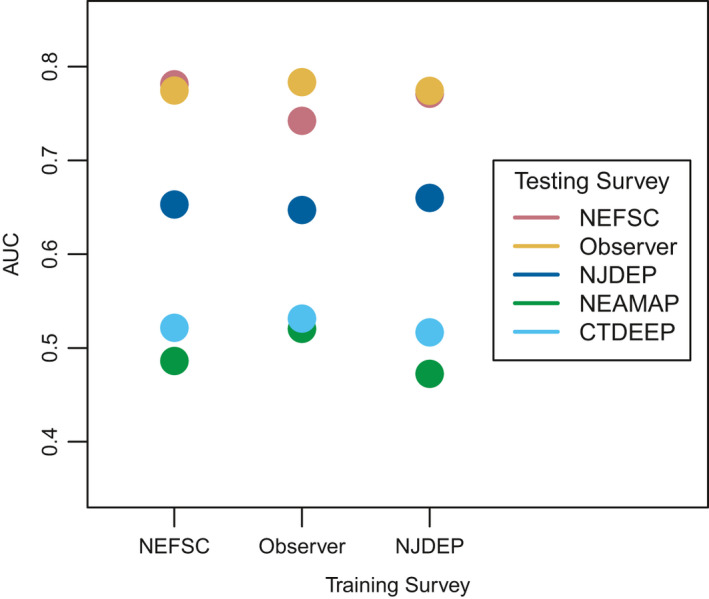
Performance, defined by AUC value, of each survey GAM when used to predict data from other available surveys

The NEFSC survey‐ and observer‐based GAMs each performed well in both the random and time‐grouped cross‐validations, with nearly every iteration having an AUC value over 0.75. The NJDEP‐based GAM also performed relatively well, though not as well as the shelf‐wide models, with the majority of iterations exceeding an AUC of 0.65 (Figure 4). Cross‐validation for predictions based on years not included in model training were comparable to random cross‐validation, but with a wider range in predictive ability (Figure 4). Shelf‐wide NEFSC and observer survey‐based GAMs were the most skillful, followed by the NJDEP‐based GAM (Figure 5). None of the GAMs were successful at predicting presence–absence in the NEAMAP or CTDEEP data.

**FIGURE 6:**
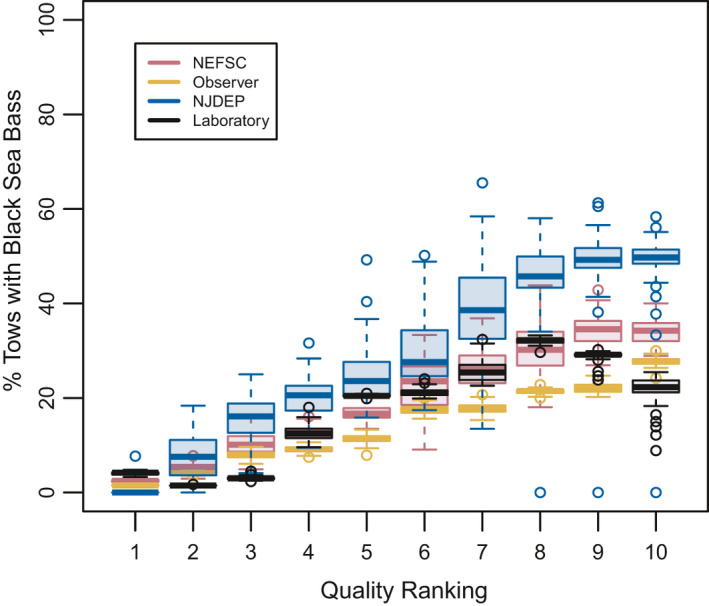
Habitat quality ranking cross‐validation for the three surveys with well‐performing GAMs (NEFSC, NJDEP, and observer) and the laboratory‐based GAM, as shown in Figure 3. Good performance is indicated by increasing percentage of tows containing black sea bass with increasing quality ranking, regardless of the total scale of the percentage

The three surveys (NEFSC, Observer, NJDEP) had an increasing proportion of tows containing black sea bass with increasing habitat quality ranking, further indicating good model performance (Figure 6). The laboratory cross‐validation did not perform as well as the surveys. There was a small uptick at the lowest habitat quality ranking, which was likely due to the very gradual slope of the curve as well as a small number of black sea bass that were observed in very cold water (<5℃, scaled response between 0 and 0.1 equivalent to quality ranking 1; see Figure 3) in the observer dataset included in testing for the laboratory‐based GAM. It should also be noted that 12℃ was the coldest temperature that black sea bass metabolic rates were measured for, so inferences colder than 12℃ were based on the model generated from warmer temperatures. Excluding the minor deviation at the lowest habitat quality ranking, black sea bass presence increased steadily with habitat quality ranking for the laboratory‐derived GAM, until reaching the highest two levels where black sea bass became less prevalent. The laboratory‐derived thermal optimum, by definition the highest habitat quality ranking, was several degrees warmer than seen in any of the survey GAMs (Figures 3,6) or even frequently sampled by any of the surveys (Figures S2–S5), so the highest quality rankings for that data were shifted into temperatures black sea bass were rarely observed in the field.

## 4 DISCUSSION

Our results demonstrate that differences in survey designs and the time of year that data are collected can strongly affect final model results, and similarities and differences between models can guide uncertainty estimates and model interpretations for various applications. Specifically in our data, varying the data source used as input to a thermal habitat model yielded different model output (e.g., *T*_opt_ range 17–25℃) but led to similar lower positive temperature response bounds (~8℃). Similarities between models can improve our confidence in their use, while differences can inform uncertainty estimates. The GAM‐derived thermal habitat models had skill at predicting years not included in model training, with only a few exceptions from data sources that performed poorly overall. However, due to the intense seasonal variability in the MAB, the models that were based on fishery data that did not cover all four seasons as well as a large spatial portion of the region, both inshore and offshore waters, are unlikely to be reliable if applied to seasons not included in the training dataset. The observer data source was the only dataset that included black sea bass presence–absence data for all 12 months of the year. However, as a fisheries‐dependent data source, it is more appropriate for species distribution analyses such as our research than it would be for biomass and abundance studies that require fisheries‐independent data such as the NEFSC bottom trawl survey.

The shelf‐wide NEFSC and observer program surveys as well as the shallow‐water NJDEP survey each exhibited clear patterns for presence–absence dependent on bottom temperature. Catch from the Connecticut DEEP survey was poorly explained by bottom temperature GAMs, likely due to the isolated nature of the enclosed Long Island Sound, as well as the high volume of localized terrestrial input compared to the rest of the shelf, resulting in presence being dependent on a more complex group of features than bottom temperature alone (Vieira, 2000). The shallow‐water NEAMAP survey spanning the MAB coastline performed poorly in the GAMs as well. While not isolated like the Long Island Sound survey, the sampling design resulted in a narrow range of temperatures and bottom habitat being sampled, thus making it impractical to develop a reliable model based solely on bottom temperature. The poor performance of the model based on this survey highlights that even a survey with wide latitudinal coverage can have low predictive power based on the timing and sampling pattern of the survey. The other surveys (NEFSC, Observer, NJDEP) exhibited clear patterns based on bottom temperature alone but had a large majority of deviance remaining unexplained. Ocean variables other than temperature are clearly very important (Cullen & Guida, 2021; McHenry et al., 2019; Miller et al., 2016), but should be carefully interpreted especially when physiological effects of those features are not well‐established (Helmuth et al., 2005). Furthermore, our study only included juveniles and adults that are capable of migrating out of suboptimal water temperatures. The effects of temperature and other environmental variables on early life history stages that have more restrictive thermal constraints must be considered to achieve a more comprehensive understanding of a species ecosystem response, as well as inform possible time lags in large‐scale thermal responses (Berlinsky et al., 2004; Cowen et al., 1993; Houde, 1989).

Despite variability in the steepness of the curves and the exact location of the optimal temperature, the two shelf‐wide thermal response curves, one based on fisheries‐dependent data and the other based on fisheries‐independent data, were very similar with a gradual increase in habitat quality with temperatures up to about 18℃ followed by a rapid decrease in quality with temperatures above that. The only skillful GAM based on inshore data (NJDEP) plateaued and failed to exhibit a decrease in thermal habitat quality at high temperatures. This GAM did not perform as well as the shelf‐wide surveys even in the within‐survey cross‐validation and also had higher skill at predicting the shelf‐wide catch data than it did predicting its own data. This suggests that bottom temperature alone is a more effective predictor shelf‐wide than it is in shallow inshore waters where other features such as productivity, bottom topography, terrestrial and riverine inputs, competition, spawning dynamics, and predator–prey interactions may play a larger role (Cowen et al., 1993; Del Vecchio & Blough, 2004; Vodacek et al., 1997). Cooler temperatures may be more deterministic to predicting distribution limitations than warmer temperatures for the US Northern stock of black sea bass population, which inhabits the most northern latitude for the species, an observation supported by several previous studies (Miller et al., 2016; Sullivan & Tomasso, 2011; Younes et al., 2020) as well as this study by the fact that all three surveys displayed a remarkably similar lower thermal limit around 8.5℃. Bottom temperature in the Mid‐Atlantic Bight rarely reaches the upper thermal tolerance limit observed in laboratory studies (>25℃; Atwood et al., 2003; Slesinger et al., 2019; Sullivan & Tomasso, 2011). These undersampled temperatures in the surveys, where response variability is high, also overlap with the optimal temperature range, and predictions of future habitat are thus based on current measurements of bottom temperature that do not typically reach the future predicted temperatures under climate change scenarios (Saba et al., 2016). Models based on survey data in this region must be interpreted with caution especially at the upper end of the thermal response curve.

The warmest thermal curve was that derived from the laboratory‐based experiments, with a thermal optimum of 24.8℃ (Slesinger et al., 2019; similar laboratory‐based thermal optima observed for northern stock by Sullivan & Tomasso, 2011 and for southern stock by Atwood et al., 2003). Under laboratory conditions, fish were fed ad libitum and relieved of energy expenditure related to finding food and evading predators. Therefore, the warmer thermal optimum observed in the laboratory could be a function of increased available energetic reserves allowing higher tolerance to warmer temperatures (Clark et al., 2013). Additionally, the use of aerobic scope as an indicator to determine optimal temperatures has recently been critiqued (see Jutfelt et al., 2018). The laboratory‐derived thermal optimum should therefore be assumed to be near a maximum thermal tolerance limit, should other limiting factors in the environment (e.g., food, reproductive costs) be minimal. There may be potential flexibility for fish to acclimatize to a higher thermal optimum as ocean warming continues, especially given that the black sea bass U.S. Southern stock extends into warmer temperatures further south and into the Gulf of Mexico (Drohan et al., 2007; McCartney et al., 2013). However, the perceived lower thermal limit is likely to remain a habitat distribution limit. Additionally, physiological data encapsulates a different metric of fitness than presence–absence data, such that presence–absence data may best reflect true short‐term in situ oceanographic limits whereas the laboratory data reflects longer‐term effects on fitness (e.g., reduced reproduction) that downstream may affect the black sea bass population.

While the cross‐validation statistics show that the models perform well, indicating that they can be reliably paired with hindcasts over time periods similar to the surveys themselves, sampling in temperature‐salinity space varied substantially between seasons and surveys. The range of temperatures sampled by the NEFSC fisheries‐independent survey during the spring was lower than that sampled during the fall, resulting in a false bimodal peak in the NEFSC survey‐based thermal habitat curve that could have been smoothed out by year‐round sampling as in the NEFSC fishery‐dependent observer dataset. Because temperatures in the MAB are seasonally variable and temperatures available to a trawl survey can be extremely different depending on time of year, predicting habitat quality for seasons not included in GAM training is likely to yield flawed results. For applications considering year‐round temperatures, a GAM based on year‐round sampling, such as the observer dataset, is likely to yield more reliable results than a GAM based on the NEFSC survey that only samples during spring and fall. However, if spring and fall are the only seasons of interest—for example, if the model was being used specifically to improve interpretation of NEFSC survey results during a stock assessment—a GAM based on NEFSC data is likely a preferable option due to the random stratified nature of the survey. Applying a GAM to years that were not used in model training, on the other hand, was shown to be reliable on average when compared to random cross‐validation, though an increased variability in model performance should be taken into account if applying GAMs to projections separated from the training dataset by several decades. There is likely no “one size fits all” model, but ensembles can better inform how to interpret a given model. In some scenarios, a qualitative model may even be a more trustworthy predictor over a continuous model (e.g., responses described as high quality, neutral quality, or poor quality as opposed to a continuous scaled response).

**FIGURE 7:**
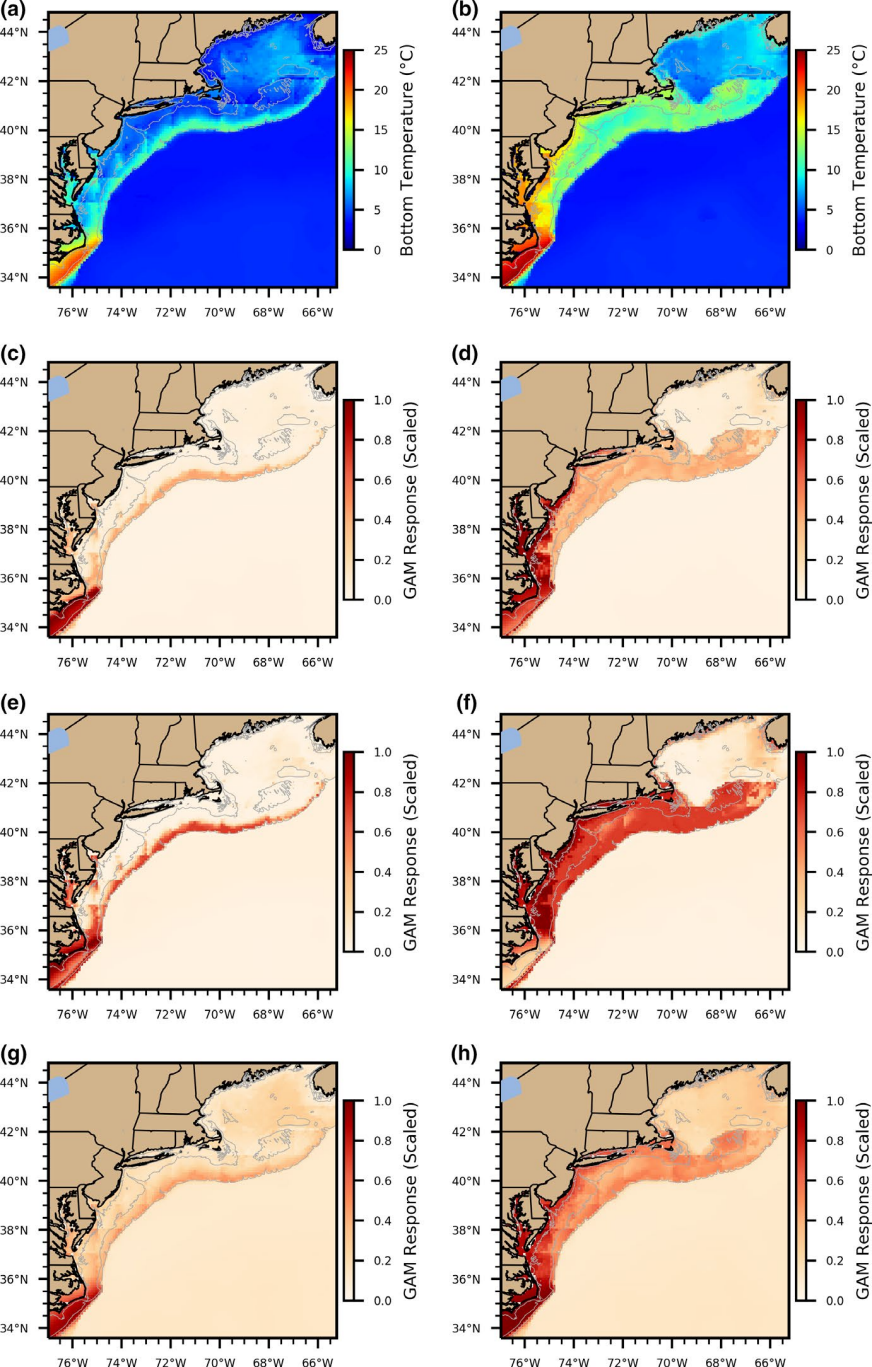
Bias‐corrected ROMS bottom temperature for spring (March–May, a) and fall (September–November, b) averaged over 1985–2015, with habitat quality as in Figure 3 projected onto those temperature fields for the NEFSC‐based GAM (c, spring; d, fall), the observer‐based GAM (e, spring; f, fall), and the laboratory‐based GAM (g, spring; h, fall)

Restricting GAMs to variables that are represented in hydrodynamic models (e.g., bottom temperature) allows for development of coupled habitat models that can have many applications, and the reliability of hydrodynamic models as they apply to niche models is supported by the similar response of in situ versus modeled temperature in this study. Thermal habitat models for various species have been coupled to ocean temperature hindcasts (Kleisner et al., 2016), to nowcasts and forecasts, and to long‐term climate change projections in order to estimate future habitat availability (Crear et al., 2020; Kleisner et al., 2017; McHenry et al., 2019; Tanaka et al., 2020). We coupled three of our GAMs (NEFSC survey‐based, observer‐based, and laboratory‐based) to seasonally averaged hindcast bottom temperature and the resulting spatial maps show that while the intensity of the spatial gradients varies, patterns of habitat quality are very similar. During the spring, habitat quality is weak across the entire shelf for all three models except for a narrow stretch of favorable habitat along the shelf break and at the southern boundary of the bight, while during the fall they each show favorable habitat across the majority of the MAB shelf and Georges Bank (Figure 7). The laboratory‐based GAM shows a much less intense spatial gradient, reflecting the gradual increase in habitat quality with temperature in cool water due to the lack of metabolic rate measurements at temperatures <12℃. Despite the strong similarities between the two shelf‐wide GAM response patterns, the observer‐based GAM predicted stronger habitat quality than predicted by the NEFSC survey‐based GAM. Thus, choosing an inappropriate model could be effective at reproducing the general spatial pattern of habitat, but may miss the strength of the spatial gradients in habitat quality or certain key patches of high‐ or low‐quality habitat. The distribution of the northern stock of black sea bass has been shifting to the north (Kleisner et al., 2016) and projections suggest that this northern shift will persist under continued ocean warming (Kleisner et al., 2017; McHenry et al., 2019), but spatial habitat modeling research must consider the uncertainty caused by fishery data used to train models particularly as it applies to habitat coupled to climate projections or other scenarios where the environment is very different from the data the habitat model were initially trained with.

GAMs are frequently used to determine the relationship between marine species and the ocean environment. However, these models often yield overfitted results that can be easily misinterpreted. To avoid this, we suggest the following: (1) consider that data source can affect the shape of the model's curve and thus the ultimate intended application of the GAM (e.g., estuary, nearshore, offshore habitat modeling); (2) GAMs coupled to hydrodynamic models should only be applied to times of year similar to those that were actually sampled by the model training data sources (e.g., applying a GAM to seasons not included in the training of the model will need to be interpreted with extreme caution); (3) an ensemble of habitat model types (e.g., GAMs, linear models, random forest, machine learning) can also be used to address single model limitations such as GAM overfitting (Tanaka et al., 2020); and (4) thermal response variability between models developed from different data sources and modeling methods can be used to inform uncertainty levels in thermal habitat predictions.

## CONFLICT OF INTEREST

None.

## AUTHOR CONTRIBUTION

**Laura Jean Nazzaro:** Conceptualization (equal); Formal analysis (equal); Investigation (equal); Methodology (equal); Software (lead); Visualization (lead); Writing‐original draft (lead); Writing‐review & editing (equal). **Emily Slesinger:** Conceptualization (equal); Investigation (equal); Methodology (equal); Writing‐original draft (equal); Writing‐review & editing (equal). **Josh Kohut:** Conceptualization (equal); Funding acquisition (equal); Methodology (equal); Supervision (equal); Writing‐original draft (equal); Writing‐review & editing (equal). **Grace Saba:** Conceptualization (equal); Funding acquisition (equal); Methodology (equal); Supervision (equal); Writing‐original draft (equal); Writing‐review & editing (equal). **Vincent S. Saba:** Conceptualization (equal); Funding acquisition (equal); Methodology (equal); Supervision (equal); Writing‐original draft (equal); Writing‐review & editing (equal).

## DATA AVAILABILITY STATEMENT

Northeast Fisheries Science Center spring and fall bottom trawl survey data were downloaded from https://oceanadapt.rutgers.edu/. All other data were obtained via email requests to the various surveys included in this analysis.

## Supporting information

Figures S1‐S6
