# Supplementary material for: Sensitivity of marine fish thermal habitat models to fishery data sources
Source: Ecol Evol. 2021 Sep 8;11(19):13001–13. doi: 10.1002/ece3.7817 (PMC8495797; doi:10.1002/ece3.7817)
Supplement: Supplementary file 1 — Figures S1‐S6 [file ECE3-11-13001-s001.docx]

Supplementary Figures: Nazzaro et al., Sensitivity of marine fish thermal habitat models to fishery data sources

Figure S1. Mean ROMS bottom temperature bias compared to 0.1° spatial resolution NOAA bottom temperature climatology for winter (December-February, a), spring (March-May, b), summer (June-August, c), and fall (September-November, d). The model was generally biased warm by about 2°C across the shelf, except for the fall when bias was lower and more spatially variable.


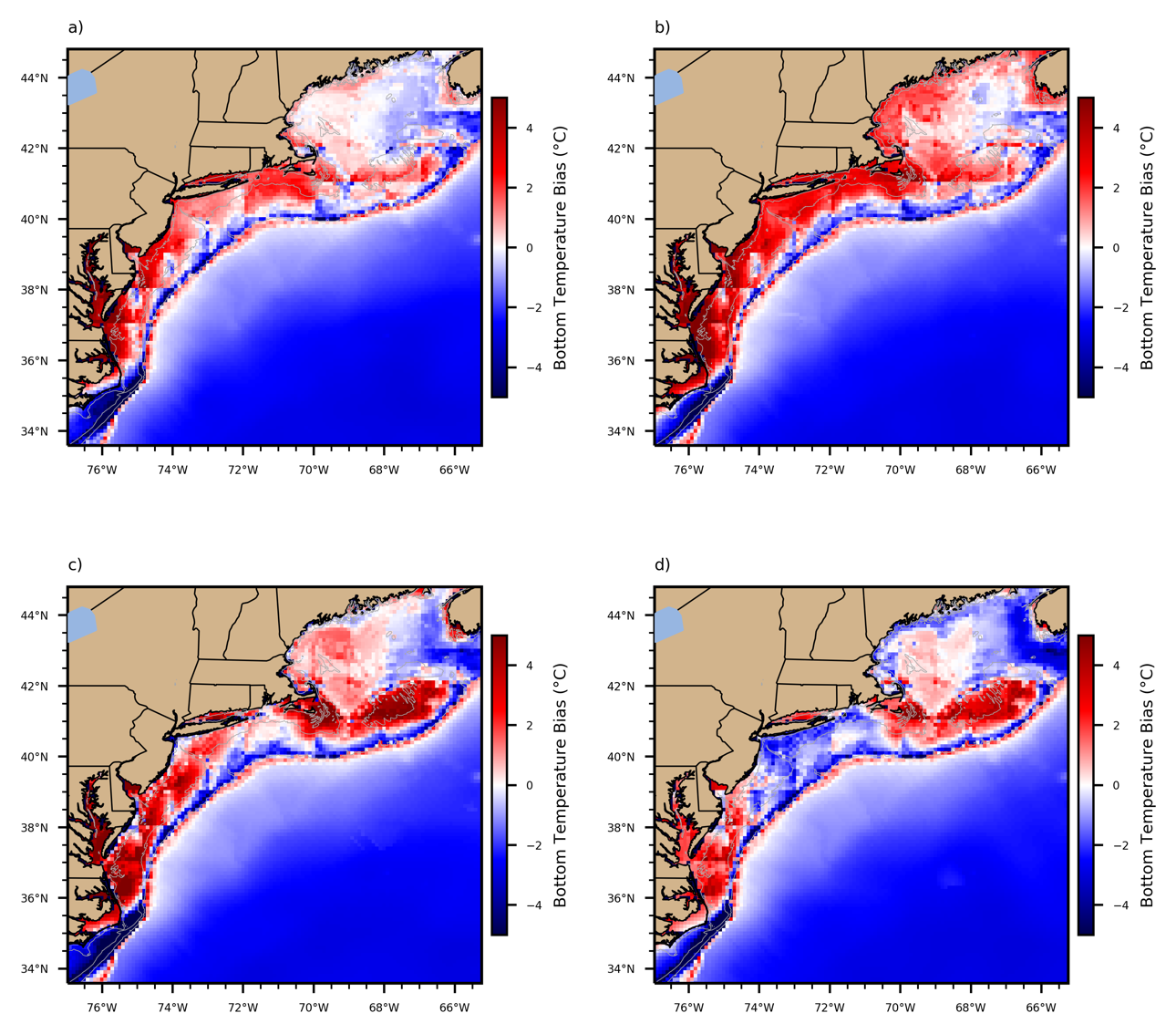


Figure S2. NEFSC spring sampling locations (a), spring sampling locations with black sea bass caught (b), fall sampling locations (c), and fall sampling locations with black sea bass caught (d), colored by date. Colormap resets between spring and fall maps but matches NEFSC maps in Figure 3. Bottom panel shows sampling in temperature-salinity space year-round (e), during spring (March-May, f), and during fall (September-November, g). Red contours mark pixels with >10 samples with black sea bass present.


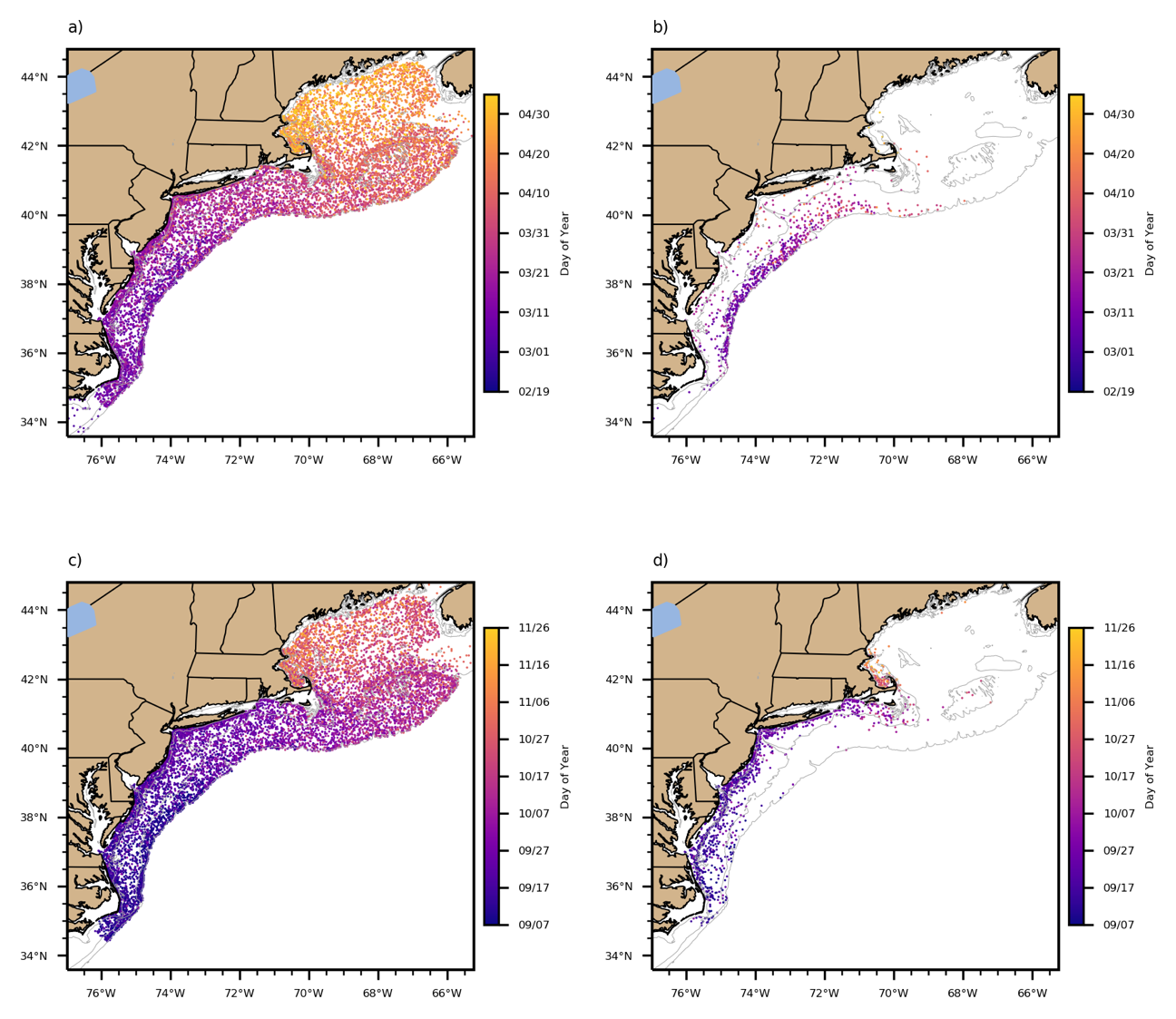

Figure S3. NEAMAP spring sampling locations (a), spring sampling locations with black sea bass caught (b), fall sampling locations (c), and fall sampling locations with black sea bass caught (d), colored by date. Colormap resets between spring and fall maps but matches NEFSC maps in Figure 2. Bottom panel shows sampling in temperature-salinity space year-round (e), during spring (March-May, f), and during fall (September-November, g). Red contours mark pixels with >8 samples with black sea bass present.


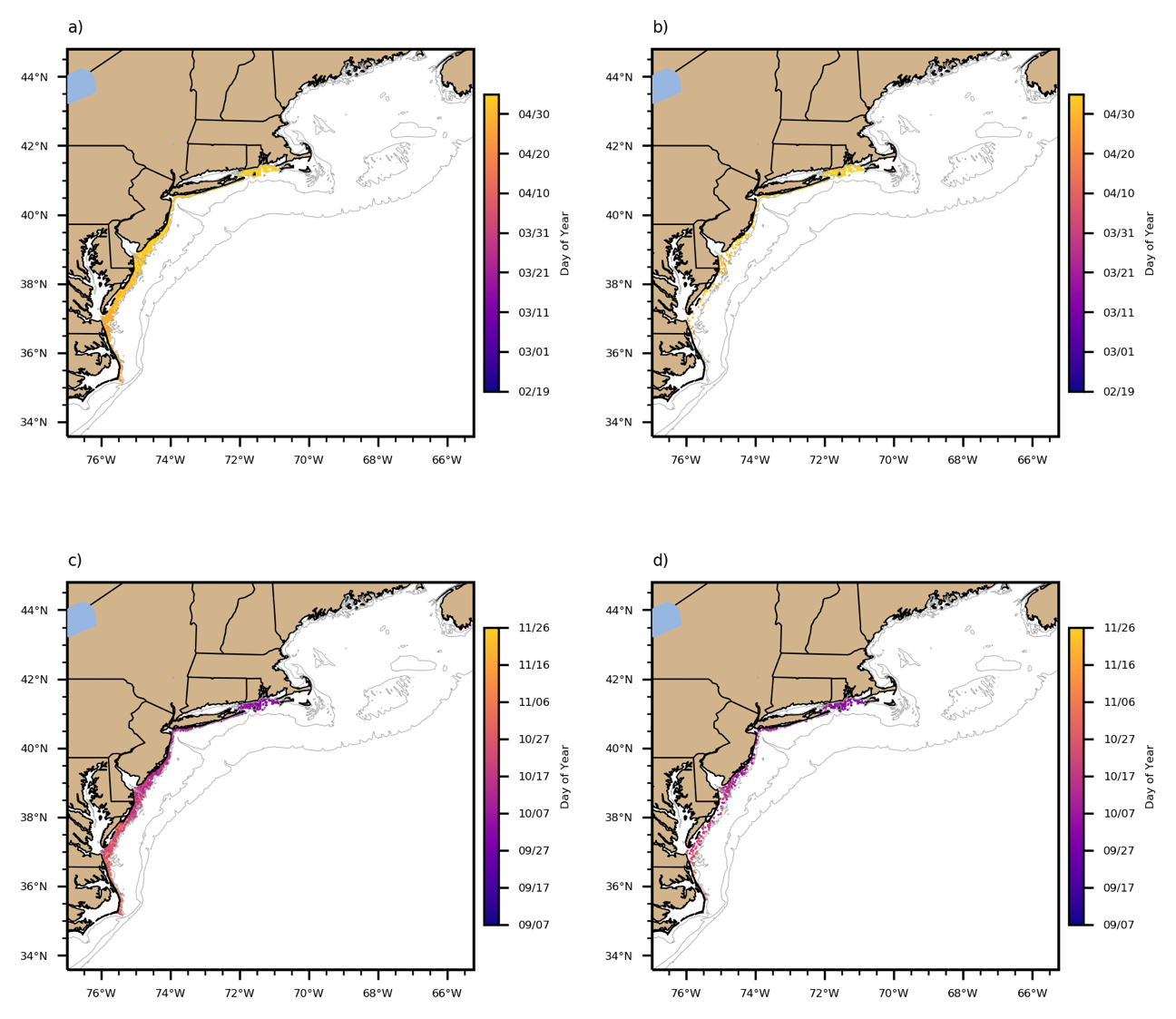

Figure S4. State survey (CTDEEP and NJDEP) sampling locations (a). Bottom panels show CTDEEP sampling in temperature-salinity space year-round (b), during spring (March-May, c), and during fall (September-November, d), red contours marking pixels with >5 samples with black sea bass present, and NJDEP sampling in temperature-salinity space year-round (e), during spring (March-May, f), and during fall (September-November, g), red contours marking pixels with >10 samples with black sea bass present. Both surveys sampled evenly throughout the survey area during each of the months listed in Table 1.


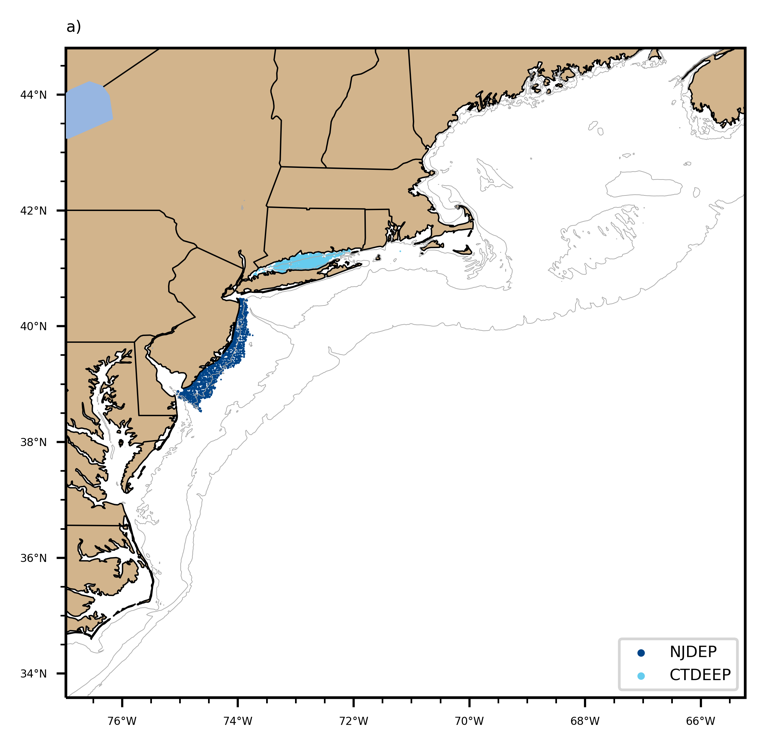

Figure S5. All federal observer sampling locations (a), and sampling locations with black sea bass present, colored by month (b). Sampling effort was fairly spatially consistent year-round.


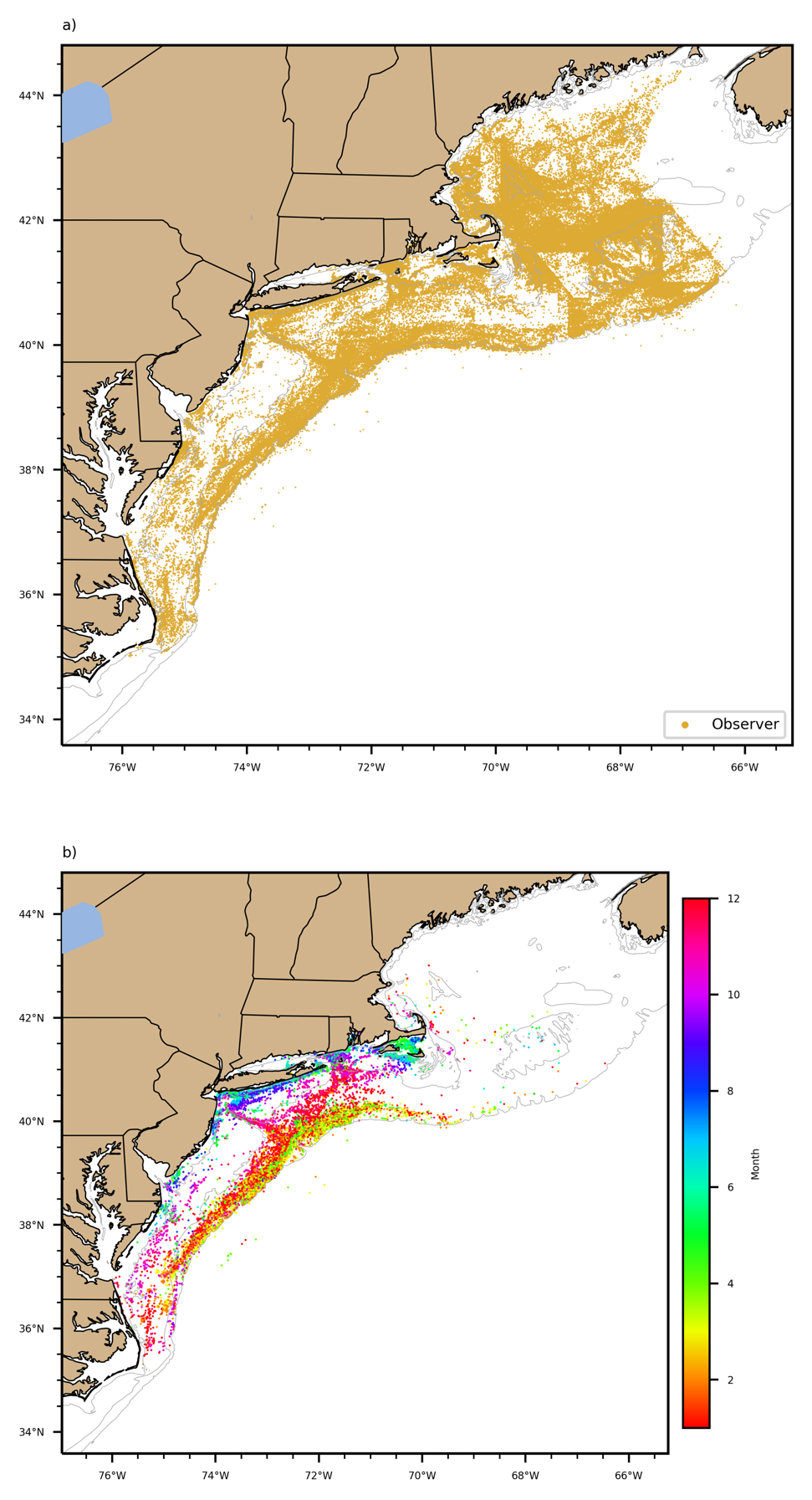


Figure S6. Bottom temperature-only partial GAM response curves from Kleisner et al. (2017) (a) and McHenry et al. (2019) (b), and GAM response based on laboratory aerobic scope data as a function of temperature from Slesinger et al. (2019) (c), +/- 1SE.
